# The role of genetics in the variable clinical response to different forms of malaria

**DOI:** 10.1186/1475-2875-13-S1-O8

**Published:** 2014-09-22

**Authors:** David Weatherall

**Affiliations:** 1 MRC Weatherall Institute of Molecular Medicine, Oxford, UK

## 

There is now strong evidence that variable degrees of protection against infection with *P. falciparum* malaria is mediated by the carrier state for a variety of different inherited disorders of haemoglobin, genetic variation in the red blood cell membrane or metabolism, and a variety of different blood groups. Similarly, genetic factors are also involved in either increasing or decreasing the susceptibility to *P. vivax* malaria. Recent work has shown that there are some remarkable epistatic interactions between the different genes involved in these conditions and the resulting susceptibility to different forms of malaria. These new findings, combined with further information obtained about the genetic basis of malaria resistance based on genome-wide association studies are already leading to a better understanding of the mechanisms whereby some of these genetic disorders offer protection to different forms of malaria, the mechanism of which is still ill-understood. In addition, these new approaches are starting to provide invaluable information about the remarkably heterogeneous distribution of some of these genetic variants, notably the inherited haemoglobin disorders.

